# Realization of Crowded Pipes Climbing Locomotion of Snake Robot Using Hybrid Force–Position Control Method

**DOI:** 10.3390/s22229016

**Published:** 2022-11-21

**Authors:** Yongdong Wang, Tetsushi Kamegawa

**Affiliations:** Graduate School of Interdisciplinary Science and Engineering in Health Systems, Okayama University, Okayama 700-8530, Japan

**Keywords:** snake robot, crowded pipes, hybrid force–position control, sinusoidal curve

## Abstract

The movement capabilities of snake robots allow them to be applied in a variety of applications. We realized a snake robot climbing in crowded pipes. In this paper, we implement a sinusoidal curve control method that allows the snake robot to move faster. The control method is composed of a hybrid force–position controller that allows the snake robot to move more stably. We conducted experiments to confirm the effectiveness of the proposed method. The experimental results show that the proposed method is stable and effective compared to the previous control method that we had implemented in the snake robot.

## 1. Introduction

Biological snakes do not have arms and legs; they use only a simple string-like body and adapt to various complex environments by appropriately changing the shape of their trunk. Hirose [1] studied biological snakes and approximated their movement patterns by a serpentine curve. By applying the locomotor properties of the biological snake to a mechanical snake robot, it is possible to develop mobile robots with high environmental adaptability by requiring only simple repetitive movement mechanisms. After Hirose’s study, many works were published on snake robots. Ma [2] investigated the form of the serpentine and proposed a more efficient meandering serpentine curve. Chirikjian [3] and Andersson [4] proposed a method to derive joint angles from approximating the robot’s configuration to the backbone curve. Yamada [5] proposed a method to derive appropriate joint angles based on the curvature and torsion of the target curve. Kamegawa [6] achieved a cylindrical climbing motion with a spiral shape by a snake robot with passive wheels. Fjerdingen [7] proposed a snake robot with an articulated transport mechanism with active wheels and joints for movement through pipe structures of different sizes and complex structures. Enner [8] developed a snake robot without wheels that could move through straight pipes of different diameters. Qi [9] enabled a snake robot to move on a pipe with branches by having a part of the snake robot’s body lift off the pipe. Takemori [10] enabled a snake robot to travel inside and outside straight pipes and outdoor trees, including those with non-circular and different cross-sections, by locally deforming the shape of the snake robot’s cross-section so that it accurately adopts the internal and external shapes. Sanfilippo [11] studied perception-driven obstacle-assisted locomotion (POAL) on the instructions between a snake robot and its environment, with virtual functional segments (VFS) along a cylindrical obstacle, and described it for the parameter settings of the cylindrical shape. Zhu [12] designed and evaluated an innovative haptic assist system for the remote operation of a snake robot for an in-pipe inspection.

In the case of climbing crowded pipes, Takemori [13] proposed a method that allows a snake robot to climb a ladder. However, it can only be applied to ladders with large spacing and requires the snake robot to be equipped with a long body length. We also proposed a method for moving a snake robot in crowded pipes [14]. The snake robot was made to form a zig-zag shape, and the snake robot achieved to move up and down, left and right, on the crowded pipes. However, it was often observed that the motors of the snake robot were reset due to an overload when we conducted the experiments. This is because the snake robot’s joint was not able to follow the target angle and generated a position deviation due to the discrepancy between the robot’s shape and the environment. Once the position deviation between the snake robot and the crowded pipes happens, this deviation may become larger and larger with the accumulation of time and eventually causes the motor to reset. Although simple position control has the advantage that it is easy to implement, it has disadvantages, such as the control requiring a high position accuracy, large torque, and low robustness. An effective solution to this problem is through the closed-loop feedback control of the robot’s target shape and torque.

In this research, we implemented a hybrid force–position control method to our snake robot. Hogan [15] pioneered the impedance control and admittance control to establish a dynamic relationship between the end-effector position and the force and to provide a unified framework for the manipulator control in free space and compliant motion in contact with the environment. Ott [16] provided a new solution for combining the impedance and admittance control by using the duty cycle as a control parameter. Fujiki [17] serially combined the admittance and impedance controllers that can adapt to unknown and variable environments in stiffness and achieve a high control accuracy and stable operation. Whitman’s [18] Shape-based Compliant (SBC) control is an example of a study that applied and implemented admittance control on a snake robot. In this paper, we implemented a hybrid force–position control method by establishing joint-level dynamics equations for our snake robot. The servo motors used in our snake robot have a function of the “current-based position control mode”. The hybrid control described in this paper is designed to take advantage of this feature. The controller consists of two parts: an impedance controller and an admittance controller. The impedance controller regulates the output force of the snake robot by using the motor angle, velocity, and acceleration information. The admittance controller regulates the motor angle based on the motor torque information. The output information of the two controllers will be output to the motor after a proportional factor regulator. The motor will then transmit the position, current, and other data to the controller to form a closed-loop control. In our previous work, we only verified the proposed controller on a simulation [19]. In this paper, based on the prior work, we have conducted comparative experiments on actual robots for the case of four controllers. The experimental results show that the proposed approach is valid. The snake robot can move efficiently in the crowded pipes environment without additional sensors and only requires feedback information sensed by the motor proprioception. The result is compared to our previous study [14], and it is observed that the snake robot traveled faster in 20 movement experiments (containing 10 upward and 10 downward directions) using the proposed method. In addition, the snake robot successfully crawled to the endpoint without any motor current reset in all the experiments.

## 2. Model Design

### 2.1. Snake Robot Model Design

**Figure 1:**
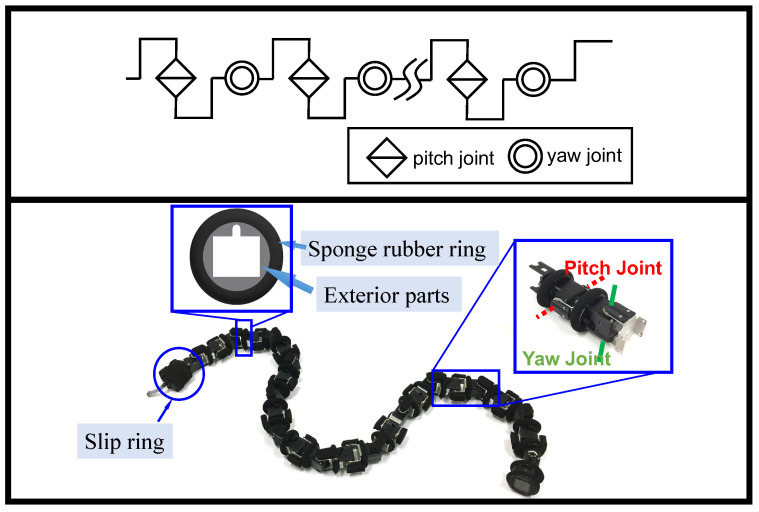
Structure of the snake robot model (**upper**) and the snake robot for the experiment (**bottom**) [14].

We use a model of a snake robot as in Figure 1, which has 2n+1 links and consists of 2n joints with $\delta_{s}$ link lengths between the joints, using a structure in which the pitch and yaw joints are interconnected alternately. The trunk radius of the snake robot is $r_{r}$, the total length is *L*, and the total weight is *m*. Let ${}^{c}\theta_{i}^{p}$ be the command *i*-th pitch joint angle, then the command joint angles of all pitch joints can be denoted by ${}^{c}\theta^{p}={\left[{}^{c}\theta_{1}^{p},{}^{c}\theta_{2}^{p},\cdots ,{}^{c}\theta_{n}^{p}\right]}^{⊤}\in R^{n}$. Similarly, let ${}^{c}\theta_{i}^{y}$ be the command *i*-th yaw joint angle, then the command joint angles of all yaw joints can be denoted by ${}^{c}\theta^{y}={\left[{}^{c}\theta_{1}^{y},{}^{c}\theta_{2}^{y},\cdots ,{}^{c}\theta_{n}^{y}\right]}^{⊤}\in R^{n}$. The command torque for each joint of the pitch and yaw joints are ${}^{c}\tau^{p}={\left[{}^{c}\tau_{1}^{p},{}^{c}\tau_{2}^{p},\cdots ,{}^{c}\tau_{n}^{p}\right]}^{⊤}\in R^{n}$ and ${}^{c}\tau^{y}={\left[{}^{c}\tau_{1}^{y},{}^{c}\tau_{2}^{y},\cdots ,{}^{c}\tau_{n}^{y}\right]}^{⊤}\in R^{n}$, respectively. Meanwhile, to facilitate the calculation, we define $P_{0}={\left[{}^{c}\theta^{p},{}^{c}\theta^{y}\right]}^{⊤}$ and $T_{0}={\left[{}^{c}\tau^{p},{}^{c}\tau^{y}\right]}^{⊤}$. The maximum output angle and the maximum output torque of the snake robot are $\theta_{max}$ and $\tau_{max}$, respectively.

### 2.2. Environment Model Design

**Figure 2:**
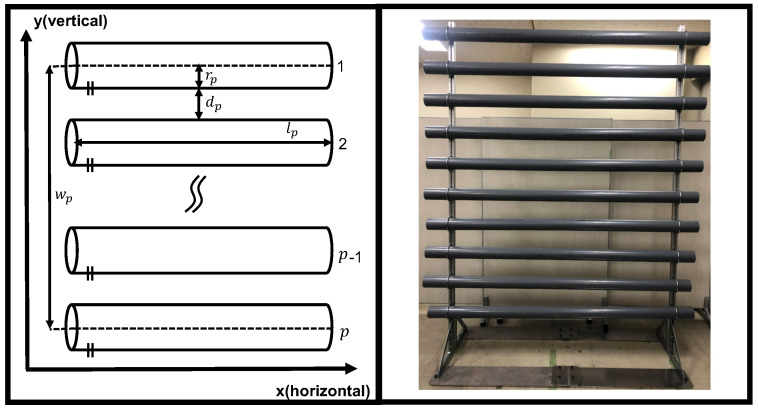
Structure of the crowded pipes model (**left**) and the crowded pipes environment for the experiment (**right**) [14].

We designed a crowded pipes model as in Figure 2. It was created by modeling the crowded pipes of an actual plant. In this model, *p* pipes of radius $r_{p}$ are arranged horizontally, and the distance between adjacent pipes is $d_{p}$. We make the direction parallel to the direction of pipes horizontal and perpendicular to the crowded pipes vertical, and the moveable distance in the vertical direction is $w_{p}$.

## 3. Control Methods

This section explains the implementation of the closed-loop hybrid force–position control method. The method is divided into five steps. The first step is to define the nominal angle of the designed curve. The second step is to define the nominal torques at different positions of the snake robot. It is assumed that the snake robot can follow the nominal angle and the nominal torque in the case of without external forces and angular differences by using the function of the servo motor. The third step is to design a force-based impedance controller to adjust the nominal torques by the angular error of the robot. The fourth step is to design a position-error-based admittance controller to adjust the nominal angle of the snake robot by the external force to which the robot is subjected. The fifth step is to design a regulator for adjusting the regulation weights of the admittance controller and the impedance controller.

### 3.1. Nominal Hybrid Force–Position Control for Crowded Pipes

#### 3.1.1. Definition of Nominal Position

Because the motion presented here is two dimensional, i.e., only pitch joints’ nominal angles are controlled by using a sine curve, while yaw joints’ nominal angles are set to zero. The following equation gives the nominal angle of the snake robot:

(1) $$\begin{array}{c} \left\{\begin{array}{cc} {}^{c}\theta^{p}= & \left[A_{\theta }\sin\left(\omega_{\lambda }\zeta_{1}-\omega_{\eta }t\right),\cdots ,\right.\\ \; & A_{\theta }\sin\left(\omega_{\lambda }\zeta_{i}-\omega_{\eta }t\right),\cdots ,\\ \; & {\left.A_{\theta }\sin\left(\omega_{\lambda }\zeta_{n}-\omega_{\eta }t\right)\right]}^{⊤}\\ {}^{c}\theta^{y}= & {\left[0,\cdots ,0\right]}^{⊤} \end{array}\right. \end{array}$$

where $\omega_{\lambda }$ is the spatial frequency of the curve, $\zeta_{i}$ is the distance from the *i*th joint to the head joint along the robot’s body, $\omega_{\eta }$ is the temporal frequency of the curve, and *t* is the departure time. The amplitude $A_{\theta }$ of the sinusoidal curve is determined by the radius of one of the crowded pipes and the radius of the snake robot. The equation is as follows:

(2) $$\begin{array}{c} A_{\theta }=r_{p}+r_{r} \end{array}$$

The spatial frequency $\omega_{\lambda }$ is determined by the length between the adjacent piping of the crowded pipes and the radius of the snake robot, which is related as follows:

(3) $$\begin{array}{c} \omega_{\lambda }=\pi /\left(2r_{p}+d_{p}\right) \end{array}$$

Substituting Equations (2) and (3) into Equation (1), we obtain the nominal angle of the snake robot.

#### 3.1.2. Definition of Nominal Torques at Different Positions

When the snake robot is controlled by the simple position control mode of the servo motor, the settings of the upper limit of the torque generated by the motor are set to the maximum value that can be set. This allows the motor to generate a large amount of torque, which is likely to cause an overload of the motor. This study proposes a new torque distribution for the snake robot moving in crowded pipes. The minimum torque appears at the vertices and inflection points of the curve, and the maximum torque appears between each minimum torque. This torque distribution aims for the robot to achieve partial softness. The nominal torque control equation is Equation (4).

(4) $$\left\{\begin{array}{cc} {}^{c}\tau^{P}= & \left[A_{\tau }\sin\left(2\omega_{\lambda }\zeta_{1}-2\omega_{\eta }t\right),\cdots ,\right.\\ \; & A_{\tau }\sin\left(2\omega_{\lambda }\zeta_{i}-2\omega_{\eta }t\right),\cdots ,\\ \; & {\left.A_{\tau }\sin\left(2\omega_{\lambda }\zeta_{n}-2\omega_{\eta }t\right)\right]}^{⊤}\\ {}^{c}\tau^{y}= & {\left[A_{\tau },\cdots ,A_{\tau }\right]}^{⊤} \end{array}\right.$$

The amplitude $A_{\tau }$ of the curve is determined by the specifications of the motor. Because the motion presented here is two dimensional, i.e., pitch joints’ nominal torques are controlled by Equation (4) using a sine curve, while yaw joint’s nominal torques are set to the maximum torque $A_{\tau }$.

### 3.2. Closed-Loop Control of Hybrid Force–Position Control

**Figure 3:**
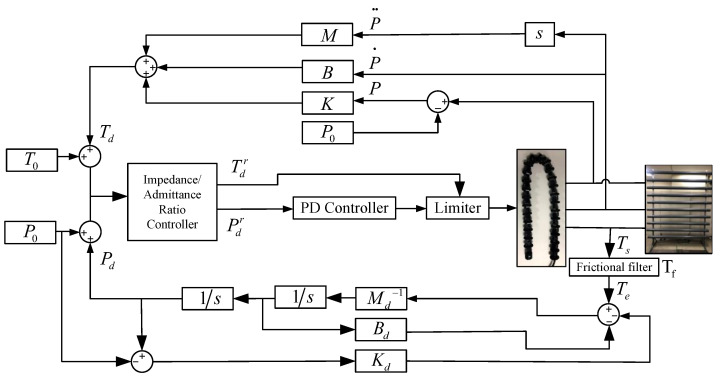
Block diagram of the system dynamics of the snake robot joint.

The closed-loop control of the force–position hybrid control consists of an impedance controller and an admittance controller. The impedance controller generates different desired torques based on the position error generated by the environment for the torque control. The admittance controller generates different desired positions based on the motor’s feedback torque for the position control. Each system is a decentralized control system at the joint level and does not use Jacobi matrices, which reduces the computational effort to some extent. A block diagram of the system dynamics for each joint is shown in Figure 3.

The meaning of each parameter of the controller is shown in Table 1. Note that the individual parameters in Table 1, although scalar, represent the control parameters of all the motors. In the following formulation of this paper, all the equations from now on are cleaned up by removing the subscript *i* in order to analyze the servo motor controller for one joint (remember that we are now dealing with the control of one servo motor, i.e., a decentralized servo motor control problem, but all the servo controllers of the snake robot use the same control strategy).

#### 3.2.1. Force-Based Impedance Control

The force-based impedance control method is shown in the upper part of Figure 3. The control system consists of a force control inside the robot and an external impedance calculation link. Based on the desired motion state of the system, the actual motion state, and the desired impedance model parameters, the external impedance controller calculates the reference adjustment force that needs to be applied to the robot joints in order to realize the desired impedance model. In addition, the specified torque is adjusted by the adjustment force so that the equivalent model for the robot and the crowded pipes is the desired impedance model. The force-based impedance control model essentially adjusts the force’s magnitude according to the robot’s deviation displacement. The equation for the impedance controller is shown as follows.

(5) $$\begin{array}{c} T_{d}=K\left(P-P_{0}\right)+B\left(\dot{P}\right)+M\left(\ddot{P}\right) \end{array}$$

#### 3.2.2. Position-Based Admittance Control

The position-based admittance control consists of an inner loop for robot position control and an outer loop for admittance control. The total torque $T_{s}$ to which the motor is subjected minus the frictional force $T_{f}$ generated by the motor is approximated as the externally applied force $T_{e}$.

(6) $$\begin{array}{c} T_{e}=T_{s}-T_{f} \end{array}$$

According to the motor characteristics used in this study, the inertia and dissipation characteristics (damping and friction) of the motor can be used to determine the friction of each motor [20]. The following equation can express the total frictional torque of the motor.

(7) $$\begin{array}{c} T_{f}=I\ddot{P}+G\dot{P}+T_{f0} \end{array}$$

where *I* is the moment of inertia of the system, *G* is the damping, $T_{f0}$ is the rotational friction of the motor. According to the external force and the desired parameter of the admittance model, the position correction is generated by the admittance controller of the control system. The reference position, the correction of the position, and the actual position are input to the position controller of the inner loop so that the actual position tracks the desired position. Thus, the robot and the crowded pipes contact action model is the desired admittance model. The equation of the admittance controller is shown as follows.

(8) $$\begin{array}{c} K_{d}\left(P_{d}-P_{0}\right)+B_{d}\left({\dot{P}}_{d}\right)+M_{d}\left({\ddot{P}}_{d}\right)=T_{e} \end{array}$$

#### 3.2.3. Impedance/Admittance Ratio Factor *r*

We designed an impedance/admittance ratio factor *r* to adjust the impedance-to-admittance ratio. Adjusting *r* adjusts the impedance and the admittance ratio of the system. The following equation expresses the adjustment torques and angles.

(9) $$\begin{array}{c} T_{d}^{r}=T_{0}+r\left(T_{d}-T_{0}\right)\\ P_{d}^{r}=P_{0}+\left(1-r\right)\left(P_{d}-P_{0}\right) \end{array}$$

The impedance/admittance ratio factor is responsible for regulating the impedance and admittance properties of the controller.

## 4. Experiments

### 4.1. Experimental Equipment

**Figure 4:**
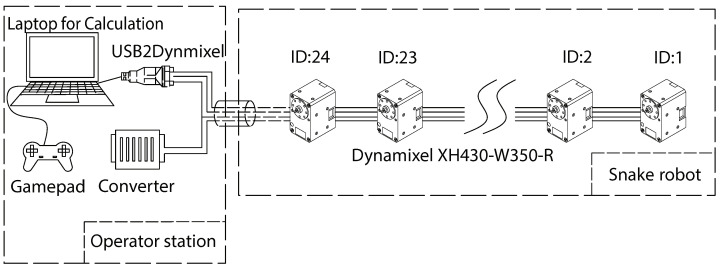
System structure of the snake robot [14].

We validate the proposed approach through the experiments conducted using an actual mechanical snake robot. Figure 4 illustrates the system structure of the snake robot. The snake robot receives 15 [V] from an external power supply. The control algorithm of the snake robot is constructed by the robot operating system (ROS), which is installed on a laptop computer. The operator provides commands to the system using a gamepad controller connected to the laptop. The snake robot and the laptop are connected via a USB2Dynamixel and the RS485 standard for the data transfer. The joints of the snake robot consist of Dynamixel XH430-W350R servo motors (Robotis Ltd., Seoul, Republic of Korea). All the motors of the snake robot are set to the position mode or the current-based position control mode. The position control mode is a closed-loop control mode that makes the actual angle track the command angle using a PID controller. The current-based position mode is an advanced setting of the Dynamixel that limits the current and supports both the position and torque (current) control. The Dynamixel can protect itself by detecting dangerous situations that could occur during the operation. When a persistent load is detected that exceeds the maximum output, an overload error will occur on the Dynamixel, and the output torque will become 0. To protect the cables of each module, the exterior part of the robot was designed to be mounted on the outside of each servo motor. In addition, the exterior part is covered with a sponge rubber ring attached to the robot’s body to reduce the impact between the robot and the crowded pipes. They also help to provide sufficient friction. The physical picture of the snake robot is shown in Figure 1; the physical picture of the crowded pipes is shown in Figure 2. The specific parameters of the snake robot and the crowded pipes are shown in Table 2.

### 4.2. Controller Setting

The individual parameters of the controller are set, as shown in Table 3. The PID controller of the servo motor is set to the PD controller, i.e., the integral controller is set to 0. The proportional gain $K_{p}^{m}$ and derivative gain $K_{d}^{m}$ of the PD controller of the servo motor, the parameter values of the controller, are determined by the trial-and-error method within the range of the values taken. We filter out the input torques below 0.5 [Nm] to remove the noise when using the admittance control to adjust the nominal position. Similarly, we filter out the input angles below 0.02 [rad] to remove the noise when adjusting the nominal torque using the impedance control.

### 4.3. Experimental Results

We have experimentally compared the performance of the proposed controller in different cases. The experiment is to move the snake robot perpendicular to the pipe. We compared the control strategies of the robot joints for the following four cases.

- The pure position control.
- The admittance control (r=0).
- The impedance control (r=1).
- The hybrid impedance/admittance control (r=0.8).

**Figure 5:**
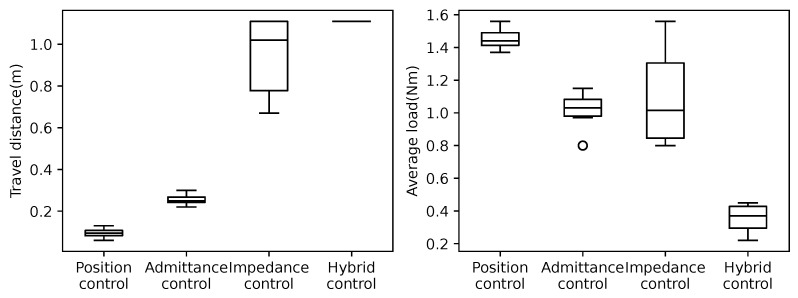
Comparison of travel distances (**left graph**) and load magnitude (**right graph**) of control methods for crawling crowded pipes upward using the snake robot. Ten trials were conducted individually for each control method. Hybrid control achieved reaching whole distance in all ten trials.

**Figure 6:**
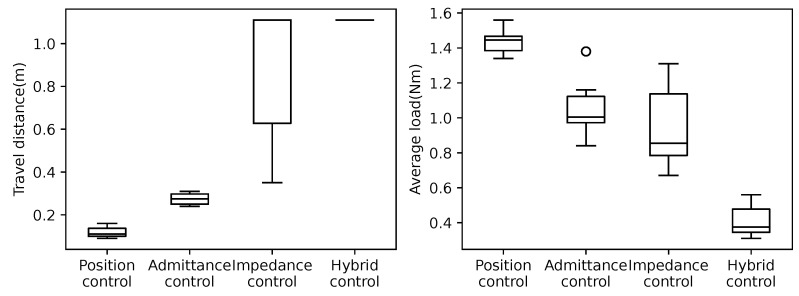
Comparison of travel distances (**left graph**) and load magnitude (**right graph**) of control methods for crawling crowded pipes downward using the snake robot. Ten trials were conducted individually for each control method. Hybrid control achieved reaching whole distance in all ten trials.

The pure position control uses the position control mode of the Dynamixel. The Dynamixel’s current-based position control mode is used in the other three controllers. The admittance control is achieved by setting the impedance/admittance ratio factor *r* to 0. Similarly, the impedance control is achieved by adjusting the impedance/admittance ratio factor *r* to 1. For the hybrid admittance/impedance control, *r* is set to 0.8 as the value determined by trial-and-error tuning. In the experiment, the robot was placed in the same initial position and moved using each control strategy. The experiment ended when the robot reached the bottom or top of the crowded pipes or when the Dynamixel detected a persistent load that exceeded the maximum output and resulted in an overload error condition. For each controller, 10 moves were conducted in the upward direction and 10 in the downward direction, for a total of 20 trials. For each position control function, the target angle is given by Equation (1) in Section 3. The parameters for the admittance control, impedance control, and hybrid impedance/admittance control are shown in Table 3, and the same values are used except for the adjustment ratio factor *r*. As the experimental results, the travel distance and motor load for the four cases moved upward and are shown in Figure 5. The travel distance and motor load of the four cases moved downward and are shown in Figure 6. Note that the motor load is the average load of all the motors. The travel distance of each case was measured by the head position of the snake robot. The maximum moving distance of the snake robot in the experiment’s environment was 1.11 [m], and the travel of 1.11 [m] indicates that the robot was able to move through the entire crowded pipes. A large variance in the travel distance indicates that the robot is not performing consistently. Additionally, a large motor load also indicates that the robot is subjected to more external forces. Moreover, a large variance in the motor load indicates that the robot is not performing consistently. Figure 5 and Figure 6 show that the hybrid control strategy always moves through all the crowded pipes, and the motor load and its variance are small compared to the other controllers.

**Figure 7:**
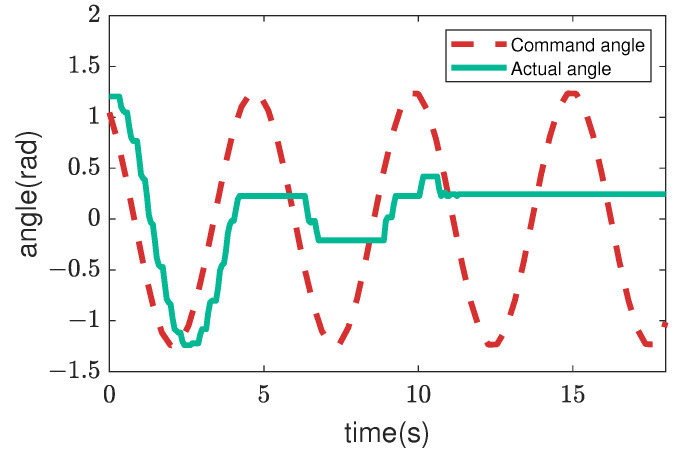
Commands and feedback data from the robot’s 3rd pitch joint are used for the trial of the pure position controller.

**Figure 8:**
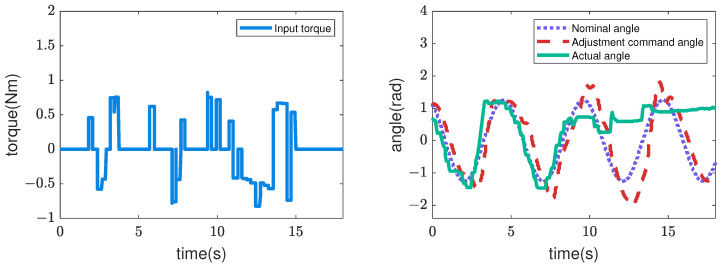
Commands and feedback data from the robot’s 3rd pitch joint are used for the trial of the admittance controller.

**Figure 9:**
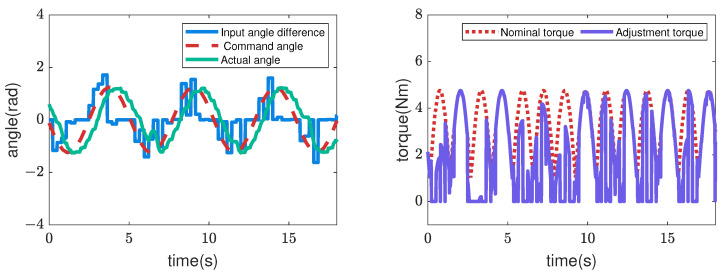
Commands and feedback data from the robot’s 3rd pitch joint are used for the trial of the impedance controller. The input angle difference was scaled 10 times for easier viewing.

**Figure 10:**
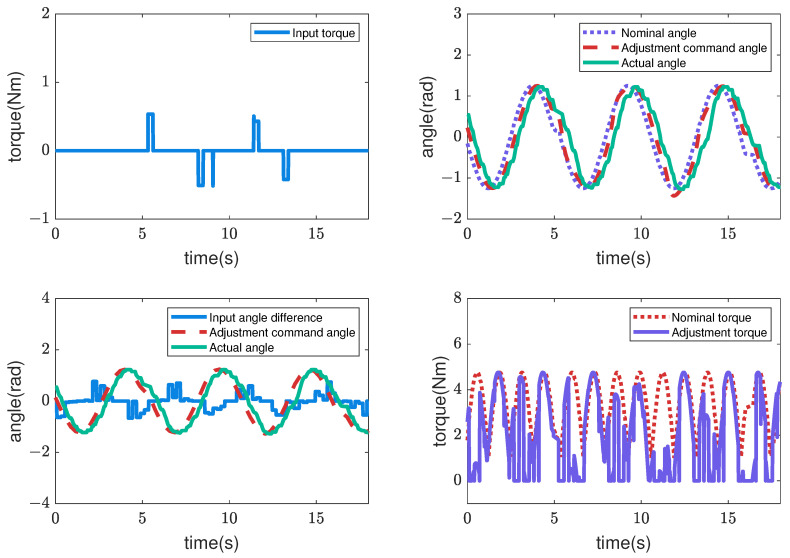
Commands and feedback data from the robot’s 3rd pitch joint are used for the trial of the hybrid controller. The input angle difference was scaled 10 times for easier viewing.

Next, Figure 7, Figure 8, Figure 9 and Figure 10 illustrate how the input changed to the third pitch joint throughout the experiment. First, the command angle and the actual angle for the pure position control case are shown in Figure 7. Figure 7 shows that the joint was driven as commanded in the first cycle, and after that, the joint was not driven to the commanded angle. As a result, the robot could hardly move through the crowded pipes. Next, Figure 8 shows the nominal angle, adjustment command angle, actual angle, and input torque when the admittance control (r=0) is used. The target angle was adjusted compared to the pure position control case of Figure 7. However, the joint received a large torque after 9 [s]. Then, the motor’s torque became zero due to the overload error at 15 [s]. Figure 5 and Figure 6 also show the loads for the 10 upward and 10 downward movements. The box plots indicate that the admittance control behavior was not performed consistently. Next, Figure 9 shows the input angle difference, command angle, actual angle, nominal torque, and adjustment torque when the impedance control (r=1) is used. Note that the input angle difference was scaled 10 times for easier viewing in Figure 9. The impedance control allowed the robot to travel further than the admittance control, and the robot sometimes reached the maximum travel distance. However, the success rate of the snake robot reaching the maximum travel distance was only 55% in the 20 trials. Finally, Figure 10 shows the input torque, nominal angle, adjustment command angle, actual angle, input angle difference, nominal torque, and adjustment torque of the robot when using the hybrid admittance/impedance control (r=0.8). Similarly, the input angle difference was scaled 10 times for easier viewing in Figure 10. The robot traveled successfully to the maximum travel distance in all 20 experiments with the hybrid control. In addition, the robot’s motors were subjected to less load than the other three controllers.

**Figure 11:**
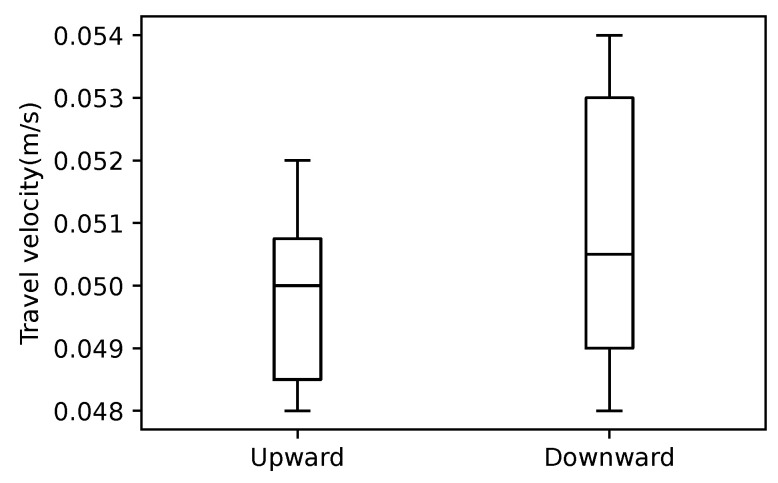
Velocity comparison of 10 upward motion experiments and 10 downward motion experiments.

**Figure 12:**
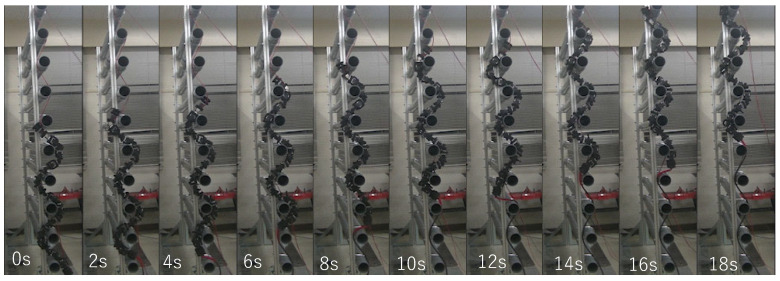
Experimental results of upward motion using hybrid force–position control method.

**Figure 13:**
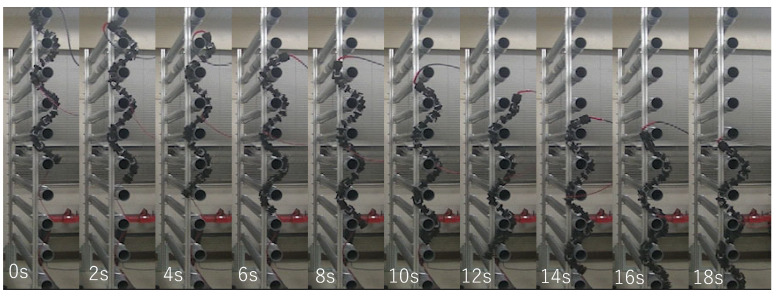
Experimental results of downward motion using hybrid force–position control method.

In addition, the travel velocity of the robot with the hybrid control is shown in Figure 11. The average values are 0.050 [m/s] and 0.051 [m/s] for the upward and the downward movement, respectively. An example of the sequential pictures of the upward and downward movements of the snake robot with the hybrid control is shown in Figure 12 and Figure 13, respectively. The travel velocity of all 20 experiments exceeded that of the literature [14], and no motor reset occurred. The results verified the effectiveness of the hybrid controller proposed in this study and solved the motor reset issue in the literature [14] when the snake robot traveled in the vertical direction.

## 5. Conclusions and Future Work

In this paper, we proposed a control method for the movement of the snake robot in the vertical direction of the crowded pipes. The proposed control method consists of a sinusoidal shape design and a hybrid controller. We illustrated the sinusoidal shape design method, described the modeling approach for the hybrid force–position control in a crowded pipes environment, programmed the proposed models, and demonstrated their effectiveness in the experiments. By comparing the four sets of experiments, we can obtain that our hybrid controller can significantly improve the crawling success rate of the snake robot and reduce the stress while crawling in the crowded pipes. In addition, the implemented hybrid controller successfully solves the issue of the motor reset due to an overload in our previous study. The future work is to experiment with crowded pipes in other settings, such as a different pipe spacing.

## Figures and Tables

**Table 1:** Meaning of each parameter of the hybrid controller.

$T_{d}$	Desired output torque	*M*	Inertia matrix for impedance control
*B*	Damping matrix for impedance control	*K*	Stiffness matrix for impedance control
$T_{0}$	Nominal output torque	$T_{d}^{r}$	Final system output torque
*P*	Present angle	$\dot{P}$	Present angular velocity
$\ddot{P}$	Present angle acceleration	*r*	Impedance/admittance ratio factor
$P_{d}$	Desired output angle	${\dot{P}}_{d}$	Desired output angle velocity
${\ddot{P}}_{d}$	Desired output acceleration	$M_{d}$	Inertia matrix for impedance control
$B_{d}$	Damping matrix for impedance control	$K_{d}$	Stiffness matrix for impedance control
$P_{0}$	Nominal output angle	$P_{d}^{r}$	Final system output angle
$T_{e}$	Total torque	$T_{s}$	External torque
$T_{f}$	Friction torque		

**Table 2:** Experimental environment setting.

*n*	12	$\delta_{s}$	60[mm]
$r_{r}$	51.00[mm]	*L*	165[mm]
*m*	3[kg]	$\theta_{max}$	1.57[rad]
$\tau_{max}$	4.8[Nm]	$r_{p}$	44.55[mm]
*p*	10	$w_{p}$	2481[mm]
$d_{p}$	132.50[mm]		

**Table 3:** Setting of each parameter of the hybrid controller.

*M*	0.1[kg]	$M_{d}$	0.4[kg]
*B*	2.0[Ns/m]	$B_{d}$	20.0[Ns/m]
*K*	2.0[N/m]	$K_{d}$	4.0[N/m]
*I*	$0.007\;[{\mathrm{Ns}}^{2}]$	*G*	0.012[Ns]
$T_{f0}$	0.006[Nm]	$K_{p}^{m}$	1.8[N/m]
$K_{d}^{m}$	0.3[Ns/m]	$\omega_{\lambda }$	15.58[rad/m]
$\omega_{\eta }$	0.87[rad/s]	$A_{\tau }$	4.8[Nm]

## Data Availability

Not applicable.
